# Revisiting macrophages in ovarian cancer microenvironment: development, function and interaction

**DOI:** 10.1007/s12032-023-01987-x

**Published:** 2023-04-11

**Authors:** Amr Ahmed El-Arabey, Samia S. Alkhalil, Samia T. Al-Shouli, Maaweya E. Awadalla, Heba W. Alhamdi, Taghreed N. Almanaa, Samah Saif Eldin M. Mohamed, Mohnad Abdalla

**Affiliations:** 1 Department of Pharmacology and Toxicology, Faculty of Pharmacy, Al-Azhar University, Cairo, 11751 Egypt; 2 Department of Clinical Laboratory Sciences, College of Applied Medical Sciences, Shaqra University, Alquwayiyah, Riyadh, Saudi Arabia; 3 Immunology Unit, Pathology Department, College of Medicine, King Saud University, Riyadh, Kingdom of Saudi Arabia; 4 Research Center, King Fahad Medical City, Riyadh, Saudi Arabia; 5 Department of Biology, College of Sciences, King Khalid University, Abha, 61413 Saudi Arabia; 6 Department of Botany and Microbiology, College of Science, King Saud University, Riyadh, Saudi Arabia; 7 Department of Clinical Laboratory science, College of Applied Sciences, Shaqra University, Alquwayiyah, Riyadh, Saudi Arabia; 8 Pediatric Research Institute, Children’s Hospital Affiliated to Shandong University, Jinan, 250022 Shandong China

**Keywords:** TAMs, Ovarian cancer, Exosome, Stem cells, Cancer associated fibroblasts, TP53

## Abstract

Tumor-associated macrophages (TAMs) are an important component of the tumor microenvironment (TME) and have been linked to immunosuppression and poor prognosis. TAMs have been shown to be harmful in ovarian cancer (OC), with a positive correlation between their high levels of tumors and poor overall patient survival. These cells are crucial in the progression and chemoresistance of OC. The primary pro-tumoral role of TAMs is the release of cytokines, chemokines, enzymes, and exosomes that directly enhance the invasion potential and chemoresistance of OC by activating their pro-survival signalling pathways. TAMs play a crucial role in the metastasis of OC in the peritoneum and ascities by assisting in spheroid formation and cancer cell adhesion to the metastatic regions. Furthermore, TAMs interact with tumor protein p53 (TP53), exosomes, and other immune cells, such as stem cells and cancer-associated fibroblasts (CAFs) to support the progression and metastasis of OC. In this review we revisit development, functions and interactions of TAMs in the TME of OC patients to highlight and shed light on challenges and excitement down the road.

## Introduction

**Fig. 1:**
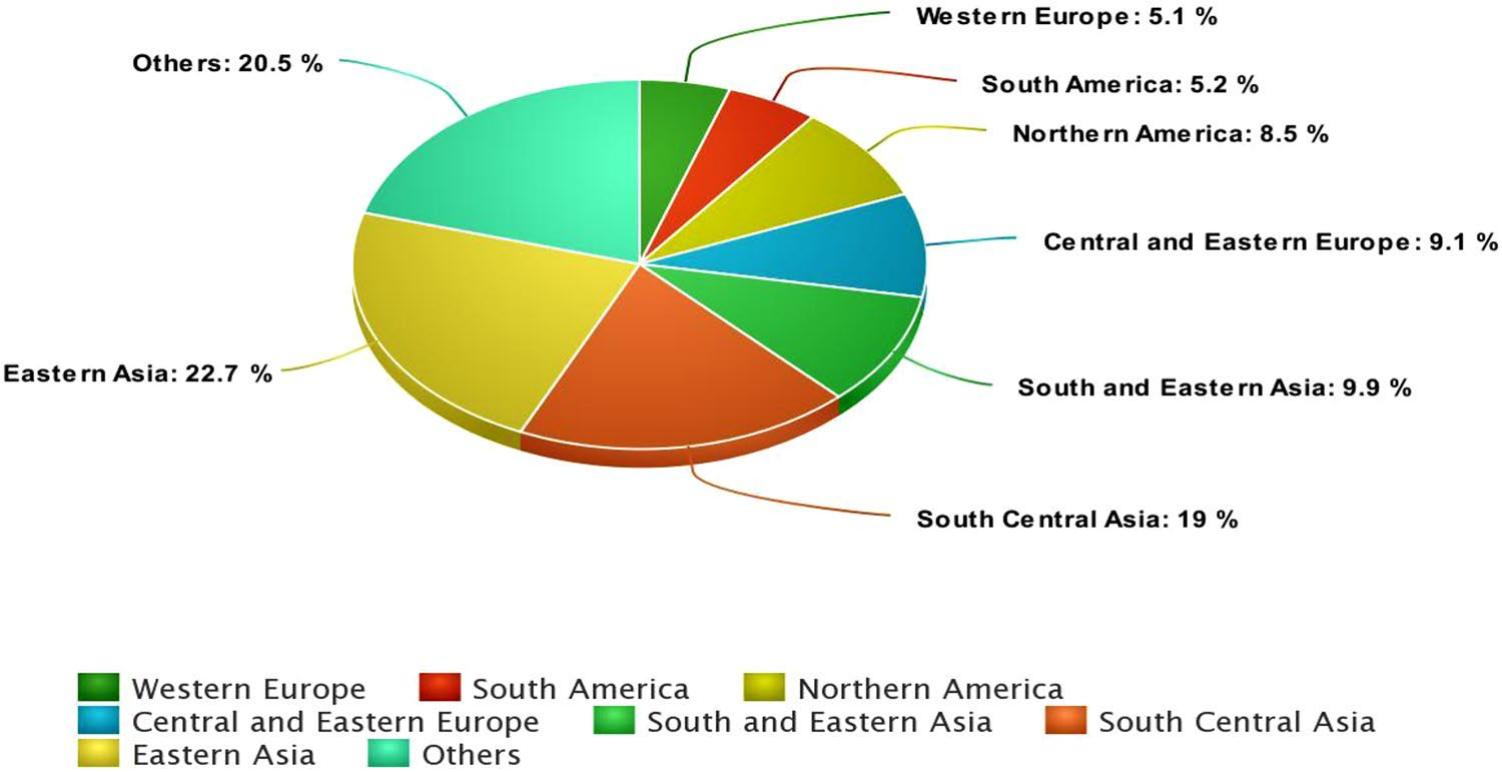
Estimated number of new cases in 2020, OC all ages: Incidence of ovarian cancer: age-standard rate (ASR) World per 100,000: (Data source: GLOBOCAN 2020; Graph production: international agency for research on cancer {IARC}-world health organization (WHO). The prevalence of OC varies greatly across the world. Eastern Asia, South Central Asia, and Central and Eastern Europe have the greatest incidence rates. Chart created with meta-chart.com

The ovaries are a pair of reproductive organs in women. They are in the pelvic region, one on each side of the uterus. Each ovary is around the size and shape of a little almond. The ovaries produce eggs as well as female hormones [1]. Pathologists categorized ovarian cancer (OC) as numerous different entities in 1930. Following that, in 1973, the world health organization (WHO) published the first systematic attempt to identify various OC subtypes [2]. Approximately 90% of OC is thought to have appeared from epithelial cell transition. As a result, the generic term for OC is epithelial OC. That terminology was intended to cover a wide range of diseases [3]. There are four histological subtypes: serous, mucinous, clear-cell, and endometrioid. Furthermore, the tumor grade assignment of OC acknowledges a higher degree of congruence for serous and endometrioid OC. Following that, the ovary’s high-grade and low-grade serous carcinomas revealed two different neoplasms with separate mechanisms of carcinogenesis, places of origin, and molecular-genetic traits. A variety of uncommon kinds, such as Brenner malignant transitional cells, as well as cases of mixed type and undifferentiated carcinoma, have also been reported [4]. Several investigations have revealed that metastatic intestinal cancers are the primary cause of many mucinous tumors. Endometriotic lesions are the cause of clear-cell and endometrioid cancer [5]. In contrast, the origin of serous carcinoma has long been contested, although in the case of High-Grade Serous Ovarian Cancer (HGSOC), it is now widely accepted that the majority of cases start in the fallopian tube [6]. In 2014, the WHO modified the classifications of the OC guidelines into two broad categories termed Type 1 and Type 2 based on molecular and genetic perspectives [7]. Type 1 subtypes include mucinous, clear-cell, low-grade serous, and brenner. It arises from pre-malignant or borderline lesions in the same way as epithelial malignancies. These cancers have wild-type Tumor Protein p53 (TP53), are genomically stable, and have frequent oncogenic alterations to many cellular signaling pathways such as RAS-Mitogen Activated Protein Kinases (MAPK) and The Phospho Inositide 3-Kinase (PI3K)-AKT Serine/Threonine Kinase. In contrast, the type 2 group, includes HGSOC, which accounts for 70–80 percent of all OC fatalities [8]. The HGSOC is distinguished by more aggressive and rapidly developing tumors, as well as an overall bad prognosis. From a molecular standpoint, these cancers are distinguished by TP53 mutations and genomic instability caused by defects in DNA repair mechanisms [6]. In the United States, epithelial OC is the main cause of gynecological cancer mortality. OC is the world’s fifth most often diagnosed cancer in women [9]. In 2021, there were an estimated 21,410 new cases and 13,770 deaths. Patients are often detected at an advanced stage because of the lack of symptoms, which may explain the low 5-year survival rate of 49.7% from 2012 to 2018 [10]. According to GLOBOCAN 2020, the incidence of OC exhibits wide geographic variations. The highest incidence rates are observed in Eastern Asia, South Central Asia, and Central and Eastern Europe. Besides, OC is the eighth most common malignancy among women [11], (Fig. 1).

The Tumor Microenvironment (TME) is a dynamic biological cellular environment surrounded by tumors that includes macrophages, stroma, stem cells, fibroblasts, lymphocytes, pericytes, adipocytes, and blood vessels [12]. The extensive, interconnected signaling networks and the particular peritoneal TME may be the main reason for the failure to successfully eliminate OC. Macrophages, T cells, Natural Killer (NK) cells, fibroblasts, and a variety of chemokines and cytokines all interact with each other to promote the development and metastasis of OC cells [13]. Macrophages are myeloid cells that play an important role in physiological homeostasis and the innate immune response. The main functions of macrophages are antigen presentation, phagocytosis, TME hemostasis, and other immunomodulatory processes [14]. They are widely known to be extremely flexible and diverse cell types characterized by low oxygen pressure, tissue necrosis, and high pyruvate and lactate concentrations [15]. Hence, bioactivity and macrophages presence influence treatments has shown promise in preclinical and clinical settings [16]. Macrophages have two phenotypes dependent on their response to certain stimuli (M1 and M2). The polarization and differentiation of macrophages reveal two unique TME subtypes: anti-tumorigenic M1 and pro-tumorigenic M2 [17]. The TME differentiates macrophages to promote M2-type macrophages, also known as Tumor Associated Macrophages (TAMs) [18]. The TAMs represent the majority of the immune cells in ascites and peritoneal macrophages, making them the most prevalent immune cell type in OC. TAMs play crucial roles in tumor initiation and progression by supporting cancer cell proliferation, angiogenesis, and lymphangiogenesis [19].

Understanding the pathobiology of OC and its distinct TME that hosts this malignancy is thus critical for developing more sensitive diagnostic tools and improved therapeutic options. Although many patients react favorably to the initial treatment, most develop chemo-resistant recurrent illnesses. Current OC therapies are still highly confined to debulking surgery and adjuvant chemotherapy. New treatment options for OC are desperately required. As a result, it is critical to comprehend not only the activity of tumor cells but also their interactions with the other components of TME [13]. Therefore, this review, will illustrate the proposed immunomodulatory functions and interactions of TAMs in the pathophysiology of OC and highlight challenges and excitements down the road based on recently published literature.

## Macrophages in epithelial ovarian cancer

**Fig. 2:**
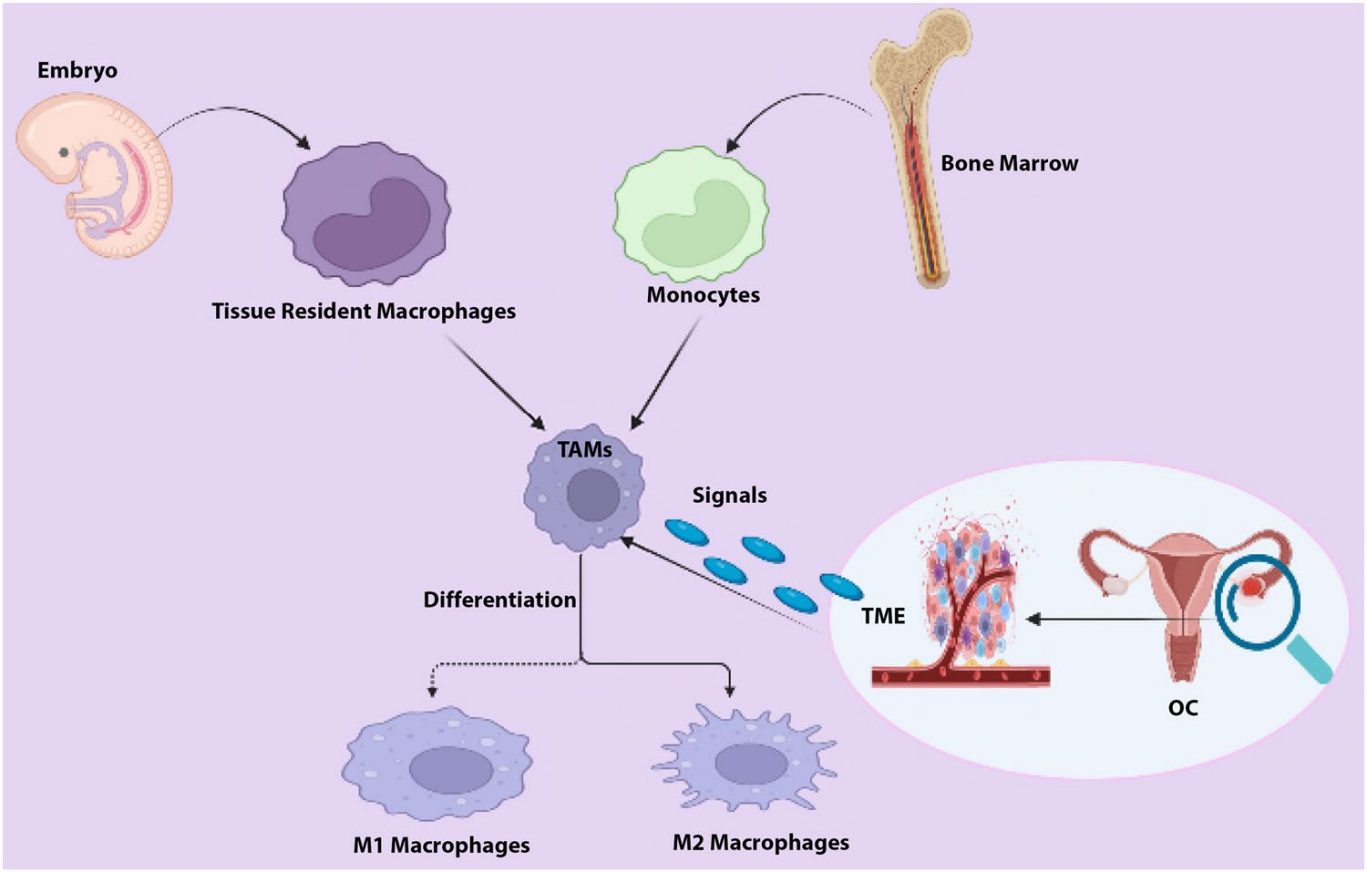
Sources of macrophages in ovarian cancer (OC): (1) Tumor-associated macrophages (TAMs) are derived from tissue-resident macrophages, which are primarily derived from the yolk sac during development, or (2) from bone marrow through monocyte differentiation. TAMs are also polarized into anti-tumorigenic M1 or pro-tumorigenic M2 phenotypes in response to tumor microenvironment (TME) of OC signals. Figure created with BioRender.com

**Fig. 3:**
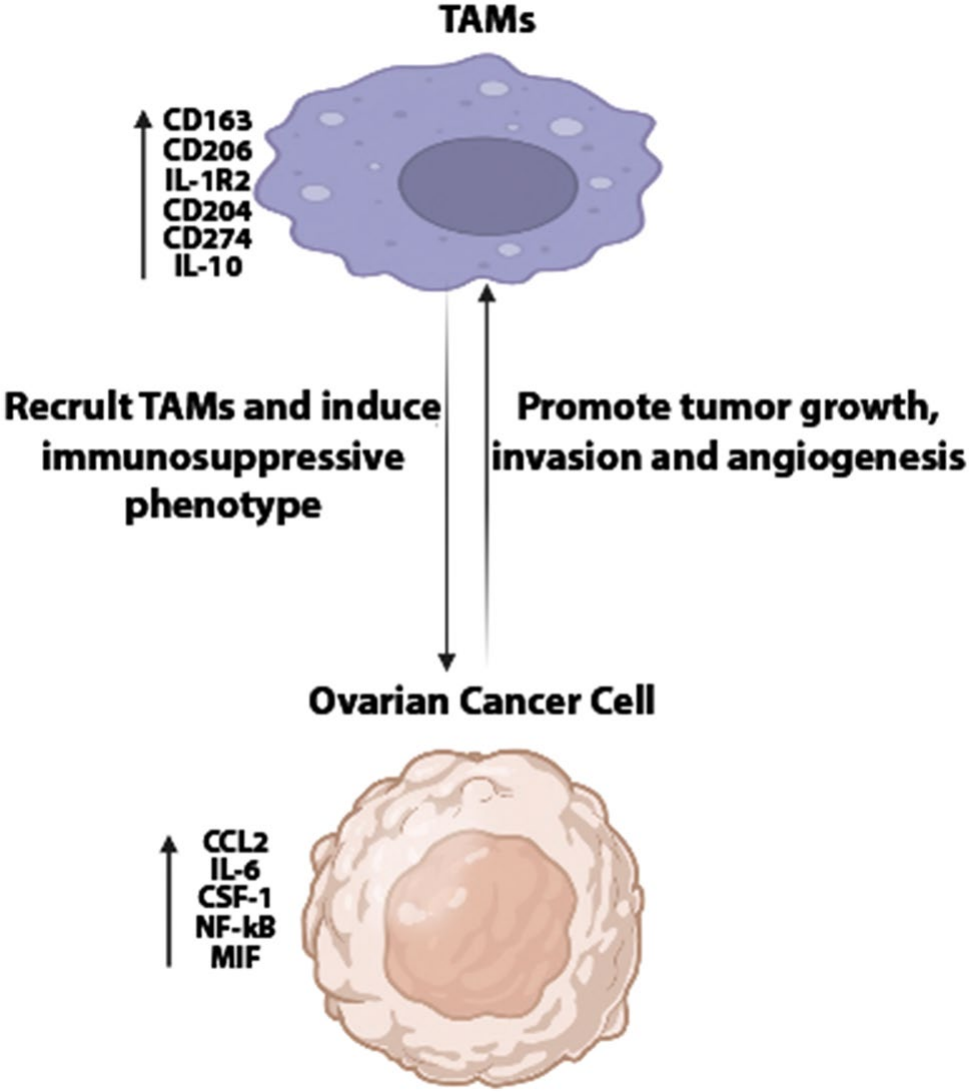
Recruitment of Tumor-associated macrophages (TAMs) into ovarian cancer (OC): OC cells produce a variety of factors (CCL2, IL-6, CSF-1, NF-κB and MIF) that attract immunosuppressive TAMs into the tumor. In addition, TAMs stimulate OC proliferation, invasion, and angiogenesis by a variety of ways dependent on TAMs marker. Figure created with BioRender.com

TAMs in OC is derived from two sources: first, long-lived resident macrophages that emerge from the embryonic yolk sac throughout development. Second, infiltrating macrophages (short-lived) that arise from bone marrow monocytes provide signals to modulate immune responses and metabolic activities in tissue-specific ways [19], (Fig. 2). Nonetheless, TAMs, have an M2-like phenotype in OC microenvironment, with high expression of Scavenger Receptor Class B (CD163), Mannose Receptor (MR, CD204) and interleukin-10 (IL-10), as well as chemokines C–C Motif Chemokine Ligand 18 (CCL18) and C–C Motif Chemokine Ligand 22 (CCL22). In contrast, the TAMs derived from ascites of OC patients are mixed polarized (M1 and M2) phenotypes [20]. The M2-like pro-tumoral TAMs are primarily engaged in OC development, metastasis, and therapeutic resistance [20]. In a mouse model of peritoneum OC, resident macrophages are significantly linked to GATA Binding Protein 6 (GATA6). Nonetheless, the peritoneum and the ascites,, which exhibit leukocyte-rich "milky patches," are invaded by OC as a result of resident macrophages [21]. Interestingly, the resident macrophages are transported to the peritoneum by retinoic acid and other inducers [22]. The the peritoneum and ascities contain a unique population of CD163^+^ Tim4^+^ resident macrophages that are important for the metastatic spread of OC cells, making them a significant premetastatic niche for the development of invasive conditions. Using genetic and pharmacological tools to selectively deplete CD163^+^ Tim4^+^ macrophages in the the peritoneum and ascities prevented tumor progression and metastatic disease spread of immortalized mouse ovarian epithelial cell line ID8. In this regard, tissue-resident macrophages play a specific role in the invasive progression of metastatic OC [23]. On the other hand, infiltrating macrophages are recruited from bone marrow monocytes to the local tissue microenvironment and differentiate further into tissue-specific macrophages, which adhere via signals from the surrounding microenvironment under homeostatic conditions. Both resident and infiltrating macrophages in the TME typically differentiate into pro-tumorigenic M2-like phenotypes in cancer [24]. The M2 (TAMs) represent both macrophage phenotypes (resident and infiltrating) and account for a significant portion of the immune cells in ascites and TME of OC. Ascites is a defining feature of OC, and presence and volume are associated with poor clinical outcomes in patients [25]. TAMs are extremely plastic cells that, depending on the stimuli, can display two distinct phenotypes: anti-tumorigenic M1-like and pro-tumorigenic M2-like [20]. Platinum drugs, such as cisplatin, can change the anticancer activity of M1 macrophages and induce migratory characteristics in OC cell lines via the CCL20-CCR6 axis [26]. Mechanistically, TAMs promote the spread of OC along the mesothelial-lined peritoneal cavity by facilitating OC cell adhesion to mesothelial cells via P-selectin overexpression [25]. The TAMs markers include mannose receptor CD206, CD163, CD204, interleukin 1 receptor type II (IL1R2), IL-10, and programmed cell death 1 ligand 1(PDL1)(CD274) [13]. However, M1 macrophages act as a subset of TAMs present in ascites, promoting the expression of interferon-gamma (IFN-γ) to induce cytotoxicity against tumor cells by Interleukin-12 (IL-12) [27]. Considerably, TAMs are known to be recruited from circulating monocytes in OC by releasing chemotactic factors such as monocyte chemoattractant protein-1 (MCP-1) (CCL2), colony stimulating factor 1 (CSF-1), interleukin-6 (IL-6), macrophage migration inhibitory factor (MIF), and Nuclear Factor kappa-light-chain-enhancer of activated B cells (NF-κB) [28], (Fig. 3). The TAMs in OC TME are pro-tumorigenic, promoting tumor growth, angiogenesis, migration, invasion, and metastasis [29]. In this sense, TAMs enhance OC metastasis by secreting epidermal growth factor (EGF), which promotes sphere formation and tumor growth [30]. The spread of cancer cells in the peritoneum is associated with an increase in the ratio of TAMs during tumor progression [31]. Clinically, high densities of cells expressing TAMs markers have been associated with poor clinical outcomes in many solid tumor types [16]. A recent study found that the status of TP53 (wild/mutant) influences macrophages infiltration in six types of cancer, including uterine carcinosarcoma (UCS), OC, low grade glioma (LGG), stomach adenocarcinoma (STAD), liver hepatocellular carcinoma (LIHC), and uterine corpus endometrial carcinoma (UCEC). However, patients with diffuse large B-cell lymphoma (DLBC), OC, mesothelioma (MESO), and STAD had poorer clinical outcomes with higher macrophages infiltration [32].

## Macrophages functions in the tumor microenvironment

Several in vitro and in vivo studies have revealed that macrophages mediate anumerous of functions, including chemotherapy resistance, by supplying soluble factors such as IL-6 and Tumor Necrosis Factor-alpha (TNF-α) to support survival signaling dependent on cathepsin B and/or S protease activity [33]. Macrophages promote invasion, migration, and chemoattractant bioavailability via EGF and CCL18 directed cytokine/chemokine release or through the protease-dependent remodeling of extra cellular matrix [34]. Besides, EGF expression is regulated by the colony stimulating factor 1 receptor (CSF-1R), the CSF-1 axis, and T cell-released Interleukin-4 (IL-4) [16]. Macrophages can regulate vascular structure by expressing TEK receptor tyrosine kinase (Tie2 receptor) to recruit vasculature via angiopoietin-2 (ANG-2) expression from mural cell/pericyte. Notably, macrophages directly stimulate angiogenesis by releasing vascular endothelial growth factor A (VEGFA) or by increasing the expression of VEGFA from endothelial cells via WNT Family Member 7B [35]. Through the expression of B7 family ligands programmed cell death 1 ligand-1 (PDL-1) and immune costimulatory protein B7-H4, macrophages directly suppress a cytotoxic T cell (CTL) response. Furthermore, indirect suppression via macrophages secreting Interleukin-10 (IL-10) to reduce dendritic cell capacity to secrete IL-12 and induce the anti-tumor immune response of TH1/CTL or recruitment of IL-10 expression via Regulatory T cells (TReg) via chemokine CCL22 [16]. Chemokines, cytokines, polypeptides, growth factors, hormones, metabolites, and matrix remodeling proteases are all produced by macrophages and have tumor-promoting properties [36]. Interestingly, some previously mentioned activities result from cell culture studies using bone marrow-derived macrophages or myeloid cell lines. Several soluble factors, including CCL2, CSF-1, MIF, IL-6, and NF-κB, were released in the case of OC [28]. Hypoxia induces an angiogenic phenotype in macrophages and in vivo by expressing low Major Histocompatibility Complex II levels. The stabilization of hypoxia inducible factor (HIF-1α) and -2α is also important in mediating macrophages pro-tumor properties. Hypoxia can cause HIF-1α-dependent lactic acid expression, which promotes arginase-1 expression in macrophages [37]. Neuropilin is the main player in recruiting macrophages (presumably MHCIILO) into hypoxic regions and supporting an immunosuppressive phenotype [38]. Emerging evidence can indicate that lymphocytes are strongly influenced by the process of macrophages polarization via the release of IL-4, IL-10, Interlukin-13 (IL-13), IFN-γ, TNF-α, and immunoglobulins [34]. Moreover, the T helper cells (CD4 + T cell) diminish response to cytotoxic therapy by altering macrophages polarization via IL-4 expression [39]. In addition, B cells have a different mechanism for macrophage polarization, which aids in the regulation of cancer inflammation [40].

## Macrophages interactions

### Macrophages and stemness

GATA transcription factor family members feature zinc-finger DNA-binding domains that bind to consensus 5′-(A/T) GATA (A/G) -3′ motifs. The GATA1-6 family members have an important function in controlling cell differentiation, proliferation, and migration. GATA-binding protein-3 (GATA-3) is the most well-known member of the GATA transcription factor family, and it coordinates the differentiation and specification of many tissues including adipose tissue, endothelial cells, kidney, hair follicles, mammary gland, parathyroid gland, nervous system, T cells, breast luminal epithelial cell, and thymocytes [9]. A number of scenarios have been developed to demonstrate the function of stem cells in cancer. Cancer stem cell (CSC) theory has been known for four decades, that tumor development resembles healthy tissue regeneration and is supported by a small number of hidden stem cells in cancer. Indeed, CSC plays an important role in tumour dormancy, adaptability, and metastasis. As a result, while chemotherapy and/or radiation treatment are effective in the majority of malignancies, many patients eventually have tumour recurrence [9, 41]. Hsiang-Ju et al. (2018) established that GATA3 is a master regulator for stemness phenotypes and HGSOC dormancy and that it may serve as a standard molecular biomarker for epigenetic treatment precision in the future The authors of this study found that GATA3 expression was greater in generated spheres from HGSOC patients’ ascites and sphere cells in HGSOC cell lines [41]. A recent study found that GATA3 is strongly expressed in HGSOC cell lines but not in the fallopian tubes, which is the primary origin of HGSOC. The Cancer Genomic Atlas (TCGA) database analysis for HGSOC patients revealed that GATA3 is much more prevalent in the TME than in the tumor purity. Besides, GATA3 is a crucial regulator in the interactions between TAMs and mutant TP53 HGSOC to enhance proliferation, motility, angiogenesis, Epithelial Mesenchymal Transition (EMT), and cisplatin resistance [13]. Nonetheless, GATA3 modulates macrophages polarization to induce epigenetic regulation through the overexpression of Lysine Specific Histone Demethylase 1(LSD1). Moreover, TAMs promote EMT via the expression of Matrix Metalloproteinase-2 (MMP-2) [17]. The cancer stem biomarker doublecortin-like kinase 1(DCLK1) is included in the top 50 genes positively correlated with GATA3 in HGSOC patients. Simillarly, the expression of GATA3 and DCLK1 were upregulated in the individual stages of HGSOC and associated with poor prognosis in HGSOC patients. DCLK1, unlike GATA3, is expressed in the normal fallopian tubes. Hence, GATA3 acts as a master regulator of HGSOC stemness via DCLK1 [9]. Furthermore, GATA3 is a promising potential target for HGSOC therapy [9].

### Macrophages and exosomes

Extracellular vesicles (EVs) have become the subject of increased study attention due to their various activities in physiology and disease. Exosomes, micro-vesicles, and apoptotic bodies are some of the subtypes of EVs based on their size and origin. Exosomes are 30–150 nm endocytic vesicles generated in the late endosomes that play significant roles in cancer biology by allowing cell-to-cell communication through the transfer of proteins, nucleic acids, and lipids [42]. Exosomes are generated by the inward budding of the membrane of the restricted multivesicular body (MVB). Late endosomal membrane invagination results in the production of intraluminal vesicles (ILVs) within big MVBs. Certain proteins are incorporated into the invaginating membrane during this process, whereas cytosolic components are engulfed and confined within ILVs. The bulk of ILVs that fuse with the plasma membrane are released into the extracellular environment as “exosomes” [43]. The “seed and soil” idea depicts the interaction between cancer cells (the seeds) and TME (the soil), as well as how the “seeds” adapt to their “soil” [44]. Exosomes, tiny endosome-derived vesicles, play an important function in TME cross-talk to promote plasticity in combination with intracellular cell trafficking [43]. Exosomes are known to convey a variety of cellular components such as enzymes, nucleic acids, transcription factors, transmembrane proteins, cytoskeleton components, and signal transducers, however the exact mechanism remains unknown. As a result, exosomes have the potential to be employed as direct therapeutic targets, biomarkers, and tailored nanocarriers. Furthermore, some studies show that exosomes from cancer cells or other cells in TME might contribute to tumor development as well as cancer therapy failure by facilitating critical interactions between diverse cell types in TME [45, 46]. Several studies deduced that exosomes derived from hypoxic OC promote macrophages M2 polarization. The process through which macrophages create diverse functional phenotypes in response to unique microenvironmental stimuli and signals is referred to as polarization [46]. Fatty acid oxidation (FAO) is an important source of energy for M2 macrophages and polarization. The IL-4 promotes macrophages FAO via signal transducer and activator of transcription 6 (STAT6) and peroxisome proliferator activated receptor-gamma (PPAR-γ) activation [47]. The polarization of macrophages into M2 phenotype plays a crucial role in the initiation, progression and metastasis of OC. As a result, focusing on macrophages-centered treatment in the OC microenvironment is a promising strategy [17].

Hypoxic exosome-educated TAMs increase OC cell growth and migration. Emerging evidence reported that numerous miRNAs, such as miR-21-3p, miR-125b-5p, and miR-181d-5p, were abundant in hypoxic OC-derived exosomes by comparing miRNA profiling of normoxic OC-derived exosomes with those in hypoxia using miRNA microarray. These miRNAs control macrophages M2 polarization via the Suppressor Of Cytokine Signaling 4/5 (SOCS4/5)/ Signal transducer and activator of transcription 3 (STAT3) pathway [48] Besides, exosomal miR-940 in OC is elevated in hypoxia and causes macrophages M2 polarization in vitro [49]. Hypoxia stimulation increases macrophages M2 polarization and improves OC cell internalization of macrophages-secreted exosomes. The miR-223 in TAMs-derived exosomes reduces OC cell susceptibility to cisplatin therapy [50]. Oncogenic miR-1246 was shown to be abundant in OC exosomes. The caveolin-1 (Cav1) gene, a direct target of miR-1246, has a role in the exosomal transfer. When OC cells co-culture with macrophages, their oncogenic miR-1246 is transmitted to M2-type macrophages but not to resting M0-type macrophages. The exosomal miR-1246, was shown to confer chemoresistance in OC through targeting Cav1/ p-glycoprotein (p-gp)/M2-type macrophages axis [51]. A recent study confirmed that GATA3 was abundantly released from TAMs via exosomes, contributing to tumor development in the TME. In this regard, GATA3 serves a unique function in HGSOC immunoediting, indicating that GATA3 may acts as a prognostic marker for HGSOC and a prospective target in HGSOC therapy [52].

### Macrophages and cancer-associated fibroblast

Cancer activity is influenced by intrinsic cancer cell features like mutations and external effects like other cells in the TME. Cancer-Associated Fibroblasts (CAFs) play an important role in the TME through their interaction with cancer cells and other TME components such as the extracellular matrix and immune cells infiltration. CAFs are a fascinating druggable target that modulate treatment efficacy. CAFs are generally formed in the pancreas and liver in response to tumor-derived stimuli by tissue-resident fibroblasts and/or stellate cells. They may also be produced by mesenchymal stem cells derived from bone marrow that have been attracted to the tumor [53, 54]. Under specific conditions, CAFs can be generated from adipocytes, pericytes, and endothelial cells. Inflammation is becoming more well recognized as a cancer hallmark, and it is closely linked to stromal fibroblast reactivity. Several studies have shown that CAFs promote cancer growth and metastasis via EMT development and their interactions with TAMs [55]. TAMs are linked to a poor prognosis in HGSOC patients through inhibiting the immune system in the TME. The well-known transcriptional factor GATA3 is substantially expressed in HGSOC cell lines but not in the fallopian tube, which is the main origin of this subtype of OC. GATA3 expression has been linked to HGSOC proliferation and migration, as well as a poor prognosis in patients with HGSOC. In addition, GATA3 is a master regulator for TAMs, epigenetic control of EMT and the interactions between TAMs and mutant TP53-HGSOC to enhance proliferation, migration, angiogenesis, and cisplatin chemo-resistance [13]. CAFs abundance has been clinically related to prognosis and metastasis in many human malignancies. A recent study found that increased CAFs infiltration is associated with worse outcomes in HGSOC patients [53]. The GATA3 expression is highly linked to a greater amount of CAFs invasion. Substantially, the molecular pathways that promote CAFs reactivity and EMT in cancer cells are extremely similar since they both include redox signaling circuitries such as HIF1A. Notably, Spearman’s rank correlation coefficient for GATA3 expression and CAFs infiltration in HGSOC patients is greater than Spearman’s rank correlation coefficient for HIF1A expression and CAFs infiltration. GATA3 interacts with HIF1A to inhibit ubiquitination and proteasomal degradation under hypoxic circumstances. As a consequence, GATA3 may function as a mediator for CAF invasion as well as attracting surrounding immune cells such as TAMs to promote tumor development and metastasis in HGSOC patients. Therefore, addressing GATA3 in HGSOC patients poses an unmet medical need in the future [53].

### Macrophages and tumor protein p53

Tumor Protein p53 (TP53) is vital for oncogenesis prevention because it regulates the expression of several genes involved in apoptosis, metabolism, DNA repair, and cell cycle arrest. Furthermore, the TP53/ oncoprotein mdm2(MDM2)/myc proto-oncogene protein (c-MYC) axis works as a physiological brake for the M2 macrophages polarization (IL-4/IL-13) process via c-MYC suppression. Several investigations have revealed that bone marrow-derived macrophages have considerable endogenous TP53 activity during the natural break for macrophages polarization [56]. The macrophages infiltration was greater in OC patients with mutant TP53 (83.4%) than in wild-type TP53 (16.6%). Several studies have shown that the impact of TAMs on tumor development might vary depending on the kind of tumor and TME. Increasing data suggest that TP53 functions as a tumor suppressor in inflammatory microenvironment responses. Furthermore, TP53 mutations can shield cancer cells from interaction with TME and the immune system, promoting tumor development [32]. Several investigations have revealed that tumors with mutant TP53 shift TAMs into tumor-supporting macrophages [52, 57]. In addition, TP53 mutations cause inflammation in response to inflammatory cytokines/chemokines and infections. Downregulation of mutant TP53 makes tumor cells more susceptible to the apoptotic effects of Tumor Necrosis Factor-alpha [58, 59]. To elicit rapid immunological responses of macrophages to environmental stresses, TP53 works as a co-regulator with NF-κB. In OC patients, NF-κB promotes Interleukin-8 (IL-8) and Growth-regulated oncogene (GRO) to suppress immunological responses and shift the phenotype of macrophages from inflammatory M1 to TAMs. TP53 and NF-κB hinder each other’s capacity to stimulate gene expression through a mechanism governed by their relative levels [32, 60, 61]. Recent research found that GATA3, the master regulator of macrophages polarization, has a negative connection with TP53 in OC patients and that the interaction between TAMs and mutant TP53 OC increases GATA3 expression [52]. As a result, mutant TP53 orchestrates macrophages infiltration in OC patients, and mutant TP53 and its co-regulators are prospective therapeutic targets in the future to eliminate OC [32].

## Conclusions

**Fig. 4:**
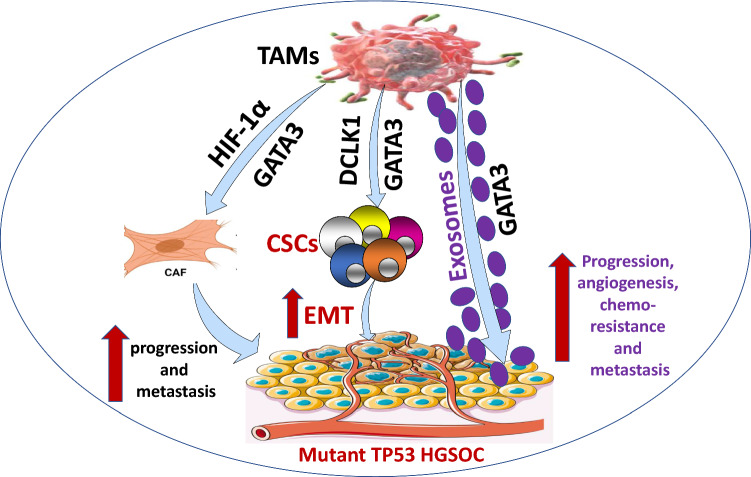
Tumor-associated macrophages (TAMs)interactions: TAMs interact with mutant TP53 of high-grade serous ovarian cancer (HGSOC) to improve the infiltration of TAMs. GATA3 was also abundantly released from TAMs via exosomes, leading to HGSOC development, angiogenesis, chemo-resistance, and metastasis. TAMs collaborate with cancer-associated fibroblasts (CAFs) to promote HGSOC development and metastasis via the GATA3-HIF-1α axis

The HGSOC is a deadly female cancer with a poor prognosis and a high rate of TAMs infiltration. TAMs have been linked to increased tumor invasiveness, metastasis, angiogenesis, chemo-resistance, and poor clinical outcomes. TAMs dominate the complicated interactions inside TME, which is a primary explanation for this clinical impact (Fig. 4). In this sense, TAMs interact with TP53, exosomes, and other immune cells, including CSC and CAFs, in the TME to support the growth and metastasis of HGSOC. Indeed, we believe that concentrating on these interactions may aid in the identification of specific OC targets. Furthermore, TAMs infiltration in HGSOC patients is mediated by TP53 status. Consequently, targeting the interactions of TAMs within TME in HGSOC patient is an appealing strategy and warrants further investigations.
